# Meta-Analysis of Cardiac Mortality in Three Cohorts of Carbon Black Production Workers

**DOI:** 10.3390/ijerph13030302

**Published:** 2016-03-09

**Authors:** Peter Morfeld, Kenneth A. Mundt, Linda D. Dell, Tom Sorahan, Robert J. McCunney

**Affiliations:** 1 Institute and Policlinic for Occupational Medicine, Environmental Medicine and Preventive Research, University of Cologne, Cologne 50937, Germany; peter.morfeld@evonik.com; 2 Institute for Occupational Epidemiology and Risk Assessment of Evonik Industries, Essen 45128, Germany; 3 Health Sciences, Ramboll Environ, Amherst, MA 01002, USA; kmundt@environcorp.com (K.A.M.); ldell@environcorp.com (L.D.D.); 4 Institute of Occupational and Environmental Medicine, University of Birmingham, Birmingham B15 2TT, UK; T.M.Sorahan@bham.ac.uk; 5 Massachusetts Institute of Technology, Cambridge, MA 02139, USA; 6 Brigham and Women’s Hospital, Boston, MA 02215, USA

**Keywords:** heart disease, ischemic heart disease, myocardial infarction, nanoparticles, occupation, epidemiology, SMR, Cox regression

## Abstract

Epidemiological studies have demonstrated associations between airborne environmental particle exposure and cardiac disease and mortality; however, few have examined such effects from poorly soluble particles of low toxicity such as manufactured carbon black (CB) particles in the work place. We combined standardised mortality ratio (SMR) and Cox proportional hazards results from cohort studies of US, UK and German CB production workers. Under a common protocol, we analysed mortality from all causes, heart disease (HD), ischemic heart disease (IHD) and acute myocardial infarction (AMI). Fixed and random effects (RE) meta-regression models were fit for employment duration, and for overall cumulative and lugged quantitative CB exposure estimates. Full cohort meta-SMRs (RE) were 1.01 (95% confidence interval (CI) 0.79–1.29) for HD; 1.02 (95% CI 0.80–1.30) for IHD, and 1.08 (95% CI 0.74–1.59) for AMI mortality. For all three outcomes, meta-SMRs were heterogeneous, increased with time since first and time since last exposure, and peaked after 25–29 or 10–14 years, respectively. Meta-Cox coefficients showed no association with lugged duration of exposure. A small but imprecise increased AMI mortality risk was suggested for cumulative exposure (RE-hazards ratio (HR) = 1.10 per 100 mg/m^3^-years; 95% CI 0.92–1.31), but not for lugged exposures. Our results do not demonstrate that airborne CB exposure increases all-cause or cardiac disease mortality.

## 1. Introduction

Epidemiological studies of exposure to airborne environmental particulates have reported associations with a variety of cardiovascular effects including myocardial infarction (MI) and ischemic heart disease (IHD) [1,2,3]. These effects were first reported among North American and European populations, and a recent study of four Chinese cities generated similar findings [4]. In light of the potential for environmental particles to increase the risk of heart disease (HD), the American Heart Association (AHA) published a position paper on particulate matter and HD, noting: “*It is the opinion of the writing group that the overall evidence is consistent with a causal relationship between PM_2.5_ exposure and cardiovascular morbidity and mortality*” [5]. A similar statement was issued by the European Society of Cardiology: “*There is abundant evidence that air pollution contributes to the risk of cardiovascular disease. Further research should explore the optimal methods of air pollution reduction and document the effects of this on the incidence of cardiovascular disease and related mortality in order to pressurize policy makers to intensify efforts required for effective legislation on air pollution reduction”* [6].

The United States Environmental Protection Agency (US EPA) also considers the association to be causal: “*Together, the collective evidence from epidemiologic, controlled human exposure, and toxicological studies is sufficient to conclude that a causal relationship exists between short-term exposures to PM_2.5_ and cardiovascular effects*” and “*Taken together, the evidence from epidemiologic and toxicological studies is sufficient to conclude that a causal relationship exists between long-term exposures to PM_2.5_ and cardiovascular effects*” [7]. 

Regulatory actions to reduce ambient particulate emissions, especially from road traffic and fossil fueled power plants, have been promulgated. For example, the US EPA implemented a 24-h limit of 35 μg/m^3^ for PM_2.5_ and a three-year annual average of 12 μg/m^3^. The US EPA also regulates PM_10_ at 150 μg/m^3^ (24 h) [8]. Similarly, the European Union requires that daily PM_10_ concentration averages must not exceed 50 μg/m^3^ on more than 35 days per year [9], and annual averages must be below 40 μg/m^3^, including those from monitoring stations near heavy automobile traffic. Low emission zones (LEZs)—areas or roads where the most polluting vehicles are restricted—have been introduced in many European cities to help meet these standards. The established associations between environmental particulate exposure and cardiac diseases and mortality served as drivers for these regulations, although the effectiveness of these measures to reduce exposures or disease has yet to be demonstrated [10,11]. 

The environmental exposure limits for airborne particulate matter (PM) in units of micrograms per cubic meter (m^3^) of air contrast sharply with occupational exposure limits for airborne particulates, which are typically orders of magnitude higher, *i.e.*, in the milligrams per m^3^ range. Notwithstanding the consistency of environmental studies demonstrating associations between particles and risk of heart diseases in the general population, studies of occupational cohorts—often at exposure levels 2–3 orders of magnitude greater than environmental concentrations—have shown small and inconsistent risks, at most [12,13]. In many of these occupational and environmental studies, however, exposures have been to complex and sometimes unknown particle and gaseous mixtures, complicating efforts to attribute risks specifically to the concentration of particles or their specific physical and chemical properties or the concomitant gaseous compounds.

To explore possible associations between airborne particle exposures and risk of heart diseases, we examined mortality from HD, IHD and AMI in three established cohorts of carbon black (CB) production workers in the United States (US) [14,15], United Kingdom (UK) [16,17], and Germany [18]. CB, a major industrial chemicals, is used primarily as a reinforcing agent for rubber products and as a pigment. In general, the CB dusts in the workplace environment occur as agglomerates or aggregates (average diameter about 80 to 500 nm for aggregates of furnace blacks, agglomerates about 1 to 100+ µm) [19] and thus, are readily inhalable [20,21]. Usually, the gravimetric method is applied to determine CB dust concentrations, *i.e.*, whatever is sampled on the filter is assumed to be essentially CB. This is a very reasonable assumption and the basis for occupational exposure limits in the US and in European countries (*vs*. analytically determining only the elemental carbon component of the dust sample). Unlike rubber manufacturing, carbon black production does not lead to mixed dust exposures and, thus, no speciation analysis is indicated. Since CB is nearly 99% pure carbon, this study offers an important research opportunity to (1) study cardiovascular disease mortality risks associated with respiratory exposure to poorly soluble particles of low toxicity; and (2) contrast findings with those of environmental studies, in which exposures are to complex mixtures of various particulates and gaseous compounds. To enhance the statistical power of our evaluation, as well as to abide by laws prohibiting the re-use and/or sharing of the individual study data, we performed meta-analyses of standardised mortality ratios (SMRs) and Cox proportional hazards results from the three studies. Our meta-analysis included 6634 workers from the US, 1147 from the UK and 1535 from Germany. This represents the largest study of CB workers, many of whom were occupationally exposed to relatively high historical concentrations of CB no longer found in these workplaces. 

## 2. Materials

We combined mortality findings from three cohorts of CB production workers. Table 6 in Dell *et al.* [15] provided a detailed comparison of the three cohorts.

### 2.1. US Cohort

The US cohort was described in Dell *et al.* [14] and Dell *et al.* [15]. Men and women known to be employed for at least one year in the period 1920–2009 at any of 18 US CB production facilities were enumerated. Most of the workers were male (88.7%), and those with unknown gender (4.5%) were treated as male. The full cohort included an inception (entry) cohort of male hourly workers hired in the periods when facility-specific employment records were complete. Mortality follow-up data were collected for the period 1940–2011 (full cohort: 6634 workers, 1947 deaths; and inception cohort: 3890 workers, 1098 deaths). We also conducted analyses in which we restricted follow-up to the period since January 1979 when the US National Death Index was initiated (full cohort: 6160 workers, 1579 deaths; inception cohort: 3360 workers, 882 deaths), as tracing deaths prior to this is known to be incomplete.

Individual work histories were collected and exposure assessment was performed as described in Dell *et al.* [15]. More than 8000 dust measurements were identified from sampling campaigns conducted between 1979 and 2007. These data were used to estimate inhalable CB concentrations in mg/m^3^. Total dust measurements were converted to inhalable dust measurements using an empirical factor of 2.97:1 (inhalable to total) derived by Kerr *et al.* [22]. Facility-specific job-exposure matrices (JEMs) were developed for five similar exposure groups (administration, laboratory, maintenance, production and materials handling/warehouse). Those study subjects without job titles (18%) were assigned plant average values. Interpolation and extrapolation of inhalable dust concentrations were performed for all years under study and time-dependent individual exposure variables were estimated. Duration of exposure to CB in years and cumulative exposure to inhalable CB in mg/m^3^-years were used as the exposure metrics in the meta-analyses.

### 2.2. UK Cohort

The UK cohort was described in Sorahan *et al.* [17] and Sorahan and Harrington [16]. Male manual workers were enumerated at five CB production facilities. All study subjects were hired in the period 1947–1974 and employed for at least one year. The full cohort included an inception (entry cohort) of workers hired in periods when facility-specific employment records were complete. Mortality follow-up data were available for the period 1951–2009 (full cohort: 1147 workers, 577 deaths; and inception cohort: 900 workers, 410 deaths). Sorahan *et al.* [17] described the exposure assessment. Limited individual work histories were available; and, industrial hygiene measurements from the end of the 1980s were available for Plants 1 and 4. A JEM for inhalable dust concentration in mg/m^3^ was estimated for 12 job categories and for 5- or 10-year calendar periods. Data for plant 4 were also used to estimate exposures for Plants 2, 3, and 5. Working conditions at these four plants were considered to be similar but different from plant 1. Time-dependent individual exposure estimates were derived for each plant: duration of exposure to CB in years; and cumulative exposure to inhalable CB in mg/m^3^-years. Study materials were anonymised some years ago, and now include no information on names, addresses, or National Health Service (NHS) numbers.

### 2.3. German Cohort

The German cohort was defined in Wellmann *et al.* [18], Morfeld *et al.* [23] and Morfeld *et al.* [24]. Further analyses were published in Büchte *et al.* [25], Morfeld and McCunney [26], Morfeld and McCunney [27], and Morfeld and McCunney [28]. Male manual workers were enumerated at one CB production facility: all were employed at least one year in the period 1960–1998 and only those workers who survived until January 1976 could be included in the study. First employment occurred in the period 1932–1997. An inception cohort was approximated as those hired between January 1960 and December 1998. The cohort was followed for mortality from January 1976 through December 1998 (full cohort: 1535 workers, 332 deaths; and inception cohort: 1276 workers, 218 deaths). No extension of this follow-up beyond 1998 was possible because of an interdiction by the data protection state officer (The data protection officer of North Rhine-Westphalia maintained the identification files of the study, but destroyed these files in September 2009. The main explanation provided was that the aim of the original study had been reached with a series of papers published in 2006). Wellmann *et al.* [18] and Morfeld *et al.* [24] described the exposure assessment. Individual work histories were available but no industrial hygiene data. For the meta-analysis, we used the corrected JEM B described in Morfeld *et al.* [24]. The corrected JEM B was based on expert assessments of semi-quantitative CB scores (intensity/concentration) in arbitrary units for 20 job titles and five plant departments in 1960 and 1998. Exposure units were assigned by experts as approximate concentrations of inhalable dust in mg/m^3^. Validation of exposure estimates was not possible. Interpolation and extrapolation were performed to all years under study to derive time-dependent individual exposure variables: duration of exposure to CB in years and cumulative exposure to (inhalable) CB in unit-years.

Using meta-analytical approaches, we combined the results from the three industrial cohorts available on CB production workers, as described below, to evaluate the relationship between CB exposure and mortality from all causes and from HD, specifically MI and IHD.

## 3. Methods

The associations between quantified CB exposure, including duration of exposure, and mortality from all causes and MI and IHD were analyzed in each cohort separately at their respective institutes (local study centers) using a common standardized protocol. Cohort-specific effect estimates then were combined and analyzed (by author Peter Morfeld) using meta-analyses. A pooled analysis of all cohort data was not possible due to data transfer and privacy restrictions noted above. 

The cohorts, upon which our analyses were based, were all assessed and approved by an institutional review board (IRB) in the USA and by data protection officers in Germany and the UK, respectively. Since the current meta-analysis involved assessment of aggregate data from previously published studies and not new individual data, no additional IRB assessments were deemed necessary. The USA study was approved by the ENVIRON Institutional Review Board (USDHHS/OHRP/IRB No. 1265 FWA No. 00006387) for project “Exposure Reconstruction and Updated Mortality Analysis for the US industrywide Carbon Black Mortality Cohort Study”.

Our meta-analysis is similar to that used by Raaschou-Nielsen *et al.* [29] to carry out the ESCAPE study on environmental particle exposure and lung cancer incidence, and that used by Vermeulen *et al.* [30] for a joint analysis of epidemiological studies on elemental carbon particle exposures from diesel motor exhaust and lung cancer mortality. 

### 3.1. Single Study Analyses

#### 3.1.1. Standardized Mortality Ratio (SMR) Analyses

We calculated SMRs (standardized mortality ratios) and 95% confidence intervals (95% CIs) for the following International Classification of Disease (ICD) groups in each cohort: all-cause mortality (ICD-9 001–799, E800–E999; ICD-10 A00-Y89); HD (ICD-9 410–429; US: ICD-9 410–414, 420–429); IHD (ICD-9 410–414, US: ICD-9 410–414, 429.2 and ICD-10 I20–I22, I24–I25, I51.3, I51.6); and AMI (ICD-9 410 and ICD-10 I21).

SMR analyses based on underlying cause of death were calculated using national rates (US and German cohorts: additionally state reference rates) stratified by 5 year-intervals of age and calendar time. Person years were censored at age 85 years such that no contributions were made to expected or observed numbers past this age.

SMRs and 95% CIs based on a Poisson distribution were calculated for the full and inception cohorts stratified by time since first exposure (abbreviated as “tsfe” and defined as period from hire) <5 years, 5–9 years, 10–14 years, 15–19 years, *etc.* SMRs were additionally stratified by time since cessation of exposure (abbreviated as “tsce” and defined as period since leaving employment) <1 year, 1–4 years, 5–9 years, 10–14 years, 15–19 years, *etc.* The first category (<1 year) included in-service deaths. 

#### 3.1.2. Cox Proportional Hazards Analyses

Time-dependent Cox proportional hazards regression models (“Cox analyses”) [31,32] were fitted. The basic time variable was attained age with staggered entry, that is, risk sets were constructed from age at start of follow-up until age at end of follow-up [33]. This approach is recommended in event history analysis when complex data structures include time-dependent terms [34,35,36], and given no intermediate confounders [37]. Follow-up was censored at age 85 years.

Cox analyses were performed for all three full cohorts (In addition, an exploratory analysis was conducted on the US and German inception cohorts.) on the following underlying causes of death: IHD (ICD-9 410–414, US: ICD-9 410–414, 429.2 and ICD-10 I20–I22, I24–I25, I51.3, I51.6) and AMI (ICD-9 410 and ICD-10 I21). Duration of exposure and cumulative exposure were treated as time-dependent variables in continuous form. In addition, because the cardiovascular effects of exposure to particles are more likely associated with recent than more distant past exposure [5], exposure estimates were also lugged by 5 years and by 10 years [16,27]. For example, in analyses lugged by 10 years, only cumulative exposure occurring in the 10 years prior to each age-specific risk set were considered. 

Time-dependent cumulative exposures also were evaluated as categorical variables. Unmodified exposures were categorized into <20, 20–49, 50–99, ≥100 whereas exposures lugged by 10 years were categorized into <5, 5–9, 10–19, ≥20 (always in mg/m^3^-years or unit-years).

Adjustment was done with three sets or combinations of covariables: year of birth (continuous) in the US, UK and German cohorts; year of birth (continuous) and plant group indicator in the US (Plant A, B+C, D and all others) and UK cohorts (Plant 1, 2 + 3, 4 and 5); and year of birth (continuous) and smoking status (active smoker *vs.* never smoker, ex-smoker *vs.* never smoker) in the German cohort. No smoking data were available for the US and UK cohorts. Year of birth was categorized by decade indicators in models with categorized cumulative exposure.

Because of the small percentage of women in the US cohort, no adjustment for gender was performed for the full cohort analyses and the inception cohort was restricted to men. 

We report coefficients with 95% CIs for duration of exposure to CB (per 10-years) and for cumulative exposure to inhalable CB dust (per 100 mg/m^3^-years for the US and UK and per 100 unit-years for Germany); Z scores (Z = coefficient/standard error of coefficient); and AIC (Akaike Information Criterion) [38]. AICs (AIC is calculated as 2 × (number of model parameters minus the partial log-likelihood of Cox model)) can be used to compare goodness of fit across nested or non-nested models, given identical response data. Smaller AICs indicate a better fit. AIC differences (Δ AIC) greater than 4 may point at a sub-optimal fit in comparison to the ‘‘better’’ model, whereas models with differences as large as 10 or 20 should be considered unsupported by the data [38].

### 3.2. Meta Analyses

Results from the three cohorts were combined to increase statistical power and summarized using meta-analyses to assess the impact of differences in SMRs and Cox relative risk (hazard ratio) estimates across cohorts [39]. Fixed effects (FE) meta-analyses were performed based on precision weighting [40], which, for SMRs, is comparable to adding observed and expected numbers across studies. FE analyses assume homogeneity of risk estimates across study components, although this assumption often is unrealistic: “Heterogeneity is common in meta-analyses of epidemiologic data and probably should be viewed as the expectation, rather than the rule” [41]; “Heterogeneity in meta-analysis should be expected and appropriately quantified” [42]. We tested the homogeneity of effect estimates across studies and derived measures of heterogeneity: I^2^ and Cochran’s Q with *p*_het_ [43]. I^2^ is the percentage of variation attributable to heterogeneity, Cochran’s Q is a heterogeneity chi^2^-statistic (chi^2^_het_) related to I^2^, *p*_het_ is the heterogeneity *p*-value belonging to Q (null hypothesis = homogeneity).

In addition, we performed random effects (RE) meta-analyses. These analyses do not assume homogeneity and take into account potential variability of SMR parameters and Cox relative risk parameters, accepting the potential heterogeneity of populations [44]. 

We used Stata 13 [45] and the “metan” command [43] to perform the analyses on log SMRs and Cox model coefficients, log hazard ratio estimates (Cox model coefficients from the German study were not adjusted for smoking).

We report FE and RE combined SMR estimates with 95% CIs, FE and RE combined Cox relative risk estimates (*i.e.*, hazard ratios) with 95% CIs as meta-effect estimates and measures of heterogeneity.

Simple RE meta-analyses of SMRs suffer from limitations because heterogeneity is only considered as a second level random effect [46]. Given heterogeneity, all kinds of simple summaries―fixed and random―do not account for the implied effect modification, so that a meta-regression modelling of log SMRs is indicated [47]. We fitted Random Effects (RE) meta-regression models [48] by restricted maximum likelihood [39].

We evaluated “tsfe” and “tsce” as continuous time variables defined by the arithmetic mean of SMR time periods. The basic model included a constant, study indicators, and the time variable. We extended this basic structure to an interaction model with constant, study indicators, time, and time × study indicators (*i.e.*, product or interaction terms). 

We report meta-effect estimates (meta-trend estimates) from the basic model: meta-SMR per 10 years of exposure with 95% CIs and the two-sided *p*-value for trend (*p*_trend_). In addition, we estimated *p*_study_ from the basic model, the two-sided *p*-value for differences among the offsets (study indicators). Also, we calculated *p*_interact_ from the interaction model, the two-sided *p*-value for heterogeneity of trends (interaction, modification of trend by study indicator). 

Cumulative exposure regression coefficients (Cox analyses) and their standard errors rely on the study-specific exposure assessment. We expect heterogeneity across studies. In particular, it is problematic to combine effect estimates based on cumulative exposures estimated as mg/m^3^-years (US, UK) with those based on estimated unit-years (Germany). The score Z (=coefficient/standard error of coefficient) avoids problems associated with non-comparable units. However, it does not directly measure the association between exposure and effect, but a standardized effect. The standardized effect can be used to estimate p-values of effect and heterogeneity, and the scores are not affected by the unit problem, but cannot be interpreted in terms of risk.

We complemented the FE and RE meta-analyses on Cox coefficients with FE meta-analyses on Cox model Z scores as follows (see also Sutton *et al.* [39] on “sum of Zs”). Given the null hypothesis of no effect of CB exposure, all observed scores Z_observed_ can be interpreted as realizations of random Gaussian variables with mean 0 and variance 1, *i.e.*, Z–N (0,1). If there are k independent studies the sum of Zs across studies is a Gaussian variable with mean 0 and variance k, *i.e.*, Ʃ Z–N (0,k). Because the studies differ in power, we weight the Zs by the relative number of expected cases based on national rates used in SMR analyses, *i.e.*, weights are w = expected/(Ʃ expected). It follows that the sum of weighted Zs, Ʃ w × Z, is a Gaussian variable M with M–N (0,Ʃ w^2^). M = Ʃ w × Z is a weighted mean of Zs, *i.e.*, the meta-Z. It could be argued that power differences in Z scores have already been taken into account by the standard deviation of the coefficient. Thus, we calculated unweighted meta-Z scores additionally.

We calculated a 2-sided *p*-value of effect, *p*, using the cumulative and reverse cumulative (lower and upper tail) distributions of M, appropriately evaluated at the observed mean of weighted Zs. Accordingly, we calculated a heterogeneity p-value, *p*_het_, based on the sum of the quadratic deviations of the observed weighted Zs from the observed weighted mean. We used the reverse cumulative (upper tail) distribution of a chi^2^-variable with k − 1 degrees of freedom.

## 4. Results

Twelve tables are presented: eight addressing results of the SMR analyses and four addressing results of the Cox models. Table 1, Table 2, Table 3 and Table 4 report SMR results for the full cohorts and Table 5, Table 6, Table 7 and Table 8 on the inception cohorts (with restricted follow-up in the US study). Two tables of SMR results are presented for each health outcome: the first reports SMRs with 95% CIs for each of the three studies and the second reports meta-analysis results. Table 1, Table 2, Table 5 and Table 6 report on overall and HD mortality; Table 3, Table 4, Table 7 and Table 8 on IHD and AMI mortality. Meta-SMRs with 95% CIs and heterogeneity statistics, based on fixed and random effects analyses, are given in Table 2, Table 4, Table 6 and Table 8. Table 1 and Table 3 report SMRs by time since first exposure, and Table 5 and Table 7, by time since cessation of exposure. All example calculations apply the appropriate national reference rates.

Table 9, Table 10, Table 11 and Table 12 present results from Cox models based on myocardial infarction: Table 9 examines meta-analysis of results by duration of exposure; Table 10 presents results for cumulative exposure (categorized and for each cohort reported separately); Table 11 presents results of the meta-analysis of the full cohorts; and Table 12 presents results of the meta-analysis of the inception cohorts (with restricted follow-up in the US study).

### 4.1. SMR Analyses

#### 4.1.1. Heterogeneity and Overall Findings

The joint mortality study analysis is based on 869 total observed deaths from HD (Table 1 and Table 2), including 747 IHD and 432 AMI deaths (Table 3 and Table 4). The largest number of cardiovascular deaths stems from the US study, which included the largest cohort.

The analyses revealed a substantial, often statistically significant heterogeneity in SMR estimates among the three cohorts, and was most pronounced for overall mortality. I^2^ was greater than 85% for all endpoints studied in the full cohort SMR analyses (Table 2 and Table 4) and the variation in SMRs attributed to heterogeneity was always statistically significant (Cochran’s chi^2^_het_ > 15, *p*_het_ < 0.01). This finding is mainly caused by consistently lower SMRs in the US study. The heterogeneity I^2^ was always greater than 50% when evaluating the inception cohorts (Table 6 and Table 8). Heterogeneity decreased partly after the US follow-up was restricted to the period 1979–2011 (comparison not shown by tables). Because of the clear heterogeneity demonstrated, only the RE analyses are appropriate to summarize the SMR data.

Full cohort RE-Meta-SMRs were 1.05 (obs = 2856, 95% CI 0.81–1.34) for overall mortality, 1.01 (observed [obs] = 747, 95% CI 0.79–1.29) for HD, 1.02 (obs = 747, 95% CI 0.80–1.30) for IHD, and 1.08 (obs = 432, 95% CI 0.74–1.59) for AMI (Table 2 and Table 4). When the follow-up period was restricted in the US study the Meta-SMRs were 1.06 (obs = 2488, 95% CI 0.84–1.33) for overall mortality, 1.03 (obs = 748, 95% CI 0.83–1.26) for HD, 1.04 (obs = 638, 95% CI 0.86–1.26) for IHD, and 1.13 (obs = 366, 95% CI 0.84–1.52) for AMI (data not shown in Tables).

Inception cohort RE-Meta-SMRs were 1.11 (obs = 1726, 95% CI 0.88–1.40) for overall mortality, 1.06 (obs = 511, 95% CI 0.85–1.33) for HD, 1.06 (obs = 436, 95% CI 0.84–1.33) for IHD, and 1.13 (obs = 255, 0.75–1.71) for AMI (data not shown in Tables). When we restricted the mortality follow-up to 1979–2011 in the US inception cohort we got the following RE-Meta-SMRs: 1.12 (obs = 1510; 95% CI 0.90–1.39) for overall mortality, 1.08 (obs = 448, 0.89–1.31) for HD, 1.08 (obs = 378, 95% CI 0.90–1.29) for IHD, and 1.18 (obs = 224, 95% CI 0.87–1.60) for AMI (Table 6 and Table 8).

FE analyses do not reveal other patterns although FE-SMRs are almost always lower than RE-SMRs. Notably, there is no statistically elevated SMR in any of the analyses performed on the four endpoints of interest. Any excesses are small and more pronounced for mortality from AMI within the inception cohorts. Nevertheless, the highest meta-estimate was obtained from the RE analysis on mortality from AMI in the inception cohorts when restricting the follow-up in the US component, and was not statistically significant (Table 8): meta-SMR=1.18, *p*_eff_ = 0.28. Heterogeneity, however, was pronounced with I^2^ = 79%, chi^2^_het_ = 9.4, and *p*_het_ < 0.01. 

We observed only marginal differences between SMRs based on national rates or state rates (virtually identical or marginally lower SMRs with state rates, and all differences within the estimated range of random fluctuations). Furthermore, restricting the follow-up period in the US study to the period 1979–2011 did not lead to any remarkable changes in the meta-SMRs.

#### 4.1.2. SMRs: Time Trends and Time Patterns 

Table 1, Table 2, Table 3, Table 4, Table 5, Table 6, Table 7 and Table 8 present SMRs by time since first exposure (full cohorts, Table 1, Table 2, Table 3 and Table 4) and SMRs specified by time since cessation of exposure (inception cohorts, Table 5, Table 6, Table 7 and Table 8). These examples suggest increasing SMRs across both time variables. Indeed, meta-regression analyses returned upward trends in SMRs across time since first exposure for all four endpoints in almost all regression models conducted on full and inception cohorts, with or without restricting the follow-up period in the US study. Analysis of the data in Table 1 yielded an increase in meta-SMRs for overall mortality by 16% per 10 years of time since first exposure (*p* = 0.015, 95% CI 3%, 29%). The differences between offsets (study indicators) were significant in this model (*p*_study_ = 0.0029) as could be expected from the finding of heterogeneity in SMRs for overall mortality between studies. Interestingly, the estimated upward trend showed no heterogeneity between study components (*p*_interact_ = 0.21). This finding of a homogeneous trend of SMRs across time since first exposure after adjustment for different baseline risks was confirmed in analyses of all combinations of endpoints and study groups. In contrast to the trends for overall mortality, none of the upward trends in mortality from HD, IHD, and AMI achieved statistical significance.

The meta-regressions on time since cessation of exposures suggested similar increases in meta-SMRs as described for time since first exposure. e.g., for the data on HD mortality of the full cohorts, as shown in Table 1 (but stratified by period since leaving), the increase in meta-SMRs was 18% per 10 years after leaving (*p* = 0.044, 95% CI 1%–38%). Heterogeneity was obvious for the offset of the regression model (*p*_study_ = 0.037) but there was no indication of heterogeneity in the trend estimate (*p*_interact_ = 0.72).The upward trends were no longer significant in HD mortality if the US follow-up was restricted to the period 1979–2011 or if incidence cohorts were analyzed. Table 5, Table 6, Table 7 and Table 8 present examples. 

Mortality from IHD and AMI mortality showed upward trends between 3% and 20% per 10 years after leaving, none of which were statistically significant. We detected no heterogeneity in offsets (*p*_study_ > 0.2) and trends (*p*_interact_ > 0.7) in any of these models. Meta-SMRs up to 1.43 were found in analyses of AMI mortality in the period of 25 to 29 years after first exposure (Table 4) and up to 1.62 in the period of 10 to 14 years after cessation of exposure (Table 8). However, the confidence intervals were wide and observed patterns could be explained by chance variations.

We found no heterogeneity in SMR trend estimates, especially after adjusting for baseline risks. This observation supports our approach to fit Cox hazard regression models across duration of exposure and/or cumulative exposure and to combine the Cox model trend coefficients *via* meta-analyses. SMRs peaked in the period 10 to 14 years after cessation of exposure, supporting our a priori decision to perform lugged Cox analyses, in particular for mortality from AMI. These Cox analyses may help to clarify whether the rising trends seen in SMRs across time variables might be associated with CB exposure estimates.

### 4.2. Cox Proportional Hazards Analyses

#### 4.2.1. Cox Models: Duration of Exposure

Meta-regression on Cox model coefficients found no indication of an effect of duration of exposure on mortality from IHD or AMI. After lugging durations by 5 years or 10 years the meta-coefficients were almost always negative but not significantly so. Without lugging, negligible positive associations were found with duration of exposure, but were never statistically significant. There was no heterogeneity of the trend coefficients across studies and no difference in findings from full cohorts, inception cohorts or after restricting the follow-up period of the US component. These results were not dependent on adjustment for plant in the UK and US components or on adjustment for smoking in the German cohort. 

As an example, we report in Table 9 the results from meta-analyses of mortality from AMI based on the full cohorts. The results of RE and FE meta-analyses exactly agree and show no effect of duration of exposure on the risk to die from AMI. The relative risk estimate was 1.01 (95% CI 0.92–1.11) per 10 years of duration of exposure if no lugging was applied but below 1 given a lug of 5 years or 10 years. The findings do not depend on adjustment for plant. The observation that results are identical in RE and FE analyses gives strong support to our finding of homogeneity of Cox model coefficients based on the chi^2^-statistics of heterogeneity, irrespective of whether we used the standard meta-analytical approach or the meta-Z scores (*p*_het_ > 0.5 always).

Results from Z score meta-analyses were similar to those from RE and FE coefficient analyses if the exposure variable is duration of exposure, which was expected because there are no differences in units and this exposure variable is similar across studies. In addition, we detected no heterogeneity in RE and FE analyses of coefficients. Thus, it is of interest to explore whether results from both kinds of analyses coincide. Indeed, *p*-values of effect from meta-Z scores in full cohort analyses on mortality from IHD were consistent with results from fixed and random effect models on coefficients. Both procedures marked the same analyses as statistically significant. *p*-values of heterogeneity were somewhat more pronounced (smaller) in meta-Z score analyses. This difference was no longer observed when follow-up was restricted to 1979–2011 in the US component (no indication of heterogeneity in both kinds of analyses, good agreement of *p*-values). Full cohort analyses of AMI showed *p*-values of effect from meta-Z scores that were consistent with results from fixed and random effects analyses on coefficients. *p*-values of heterogeneity were very close. Results did not change after follow-up was restricted in the US component. Thus, meta-Z score analyses are helpful secondary statistics to measure significance of effects and heterogeneity. They can be applied reliably even if the definition and/or units of exposure variables differ across studies as it is the case in this meta-analysis when cumulative exposures from the three studies are evaluated simultaneously. When we calculated meta-Z scores without weighting by number of expected cases we obtained essentially the same values. For comparison to results, e.g., on full cohorts as reported in Table 11, unweighted Meta-Z *p*-values for cumulative exposure models with adjustment for plant were *p* = 0.08, *p*_het_ = 0.27 (no lugging), *p* = 0.36, *p*_het_ = 0.87 (lug = 5 years), and *p* = 0.57, *p*_het_ = 0.90 (lug = 10 years).

#### 4.2.2. Cox models: Cumulative Exposure

Cox model results for categorized cumulative exposure and mortality from AMI are presented in Table 10, separately for each of the three full cohorts. Adjustment for plant reduced AICs considerably in the US component (Δ AIC > 12) which proves a substantially better fit after taking plant into account. In contrast, no advantage of adjustment by plant was demonstrated in the UK component (0 ≤ Δ AIC < 1). The very large AIC difference observed in the German component after adjustment for smoking habits (Δ AIC > 200) cannot be fully interpreted because smoking data were available only for a subset of the cohort. Nevertheless, we note that the German study provided no indication of a positive association between cumulative exposure and mortality from AMI regardless of adjustment for smoking. 

Lugging exposures by 10 years (Table 10) led to better fitting models in the German cohort after adjustment for smoking (Δ AIC > 6), but worse fitting models in the US component (Δ AIC < −5), and only minor differences in goodness of fit in the UK study as well as in the German study if not adjusting for smoking (0 < Δ AIC < 3).

Only the US study showed a significant association with AMI (Table 10) restricted to the highest exposure category: e.g., Cox model coefficient = 0.53 (95% CI 0.17, 0.89) for cumulative exposure ≥100 mg/m^3^-years when adjusting for plant. Fitting continuous exposure models in each cohort with no lugging, lug of 5 years or lug of 10 years, respectively, yielded trend coefficients for cumulative exposure that never achieved statistical significance and varied in sign, most likely reflecting random variation (data not shown). 

Meta-hazard ratios for mortality from AMI are reported in Table 11 for the three full cohorts. Some increase in relative risk was seen relative to the reference unit of cumulative exposure, but only when no lugging was applied: RE relative risk estimates were 1.10 or 1.05, with or without adjustment for plant (95% CI 0.92–1.31 or 0.99–1.10, respectively). This increase was not statistically significant as also shown by meta-Z analysis: *p* = 0.09 or 0.11 after adjustment for plant. Analyses demonstrated that all trend estimates were rather homogeneous across the three studies (*p*_het_ > 0.1). 

Findings were similar in incidence cohorts and when restricting the US mortality follow-up period to 1979–2011 (compare, e.g., Table 12). After adjusting for plant (which produced a better fit) the RE meta-hazard ratio was 1.05 (95% CI 0.97, 1.13) per reference unit of cumulative exposure (and with no lugging). Meta-Z = 1.12 (*p* = 0.15) supported the finding of no significant excess. We note that no excess relative risks were indicated in lugged analyses. Heterogeneity was not demonstrated in any of the analyses (*p*_het_ > 0.35).

FE and RE analyses of IHD mortality in the full cohorts resulted in a meta-hazard ratio of 1.02 per 100 mg/m^3^-years or 100 unit-years after adjustment for plant (95% CI 0.98–1.06, *p*_het_ >0.4). After lugging no increase of relative risks across cumulative exposure was found regardless of adjusting for plant. Overall, regression model findings and meta-results on mortality from IHD were similar to those from AMI (data not shown). 

### 4.3. Summary of Results

Full cohort RE Meta-SMRs were 1.01 (obs = 747, 95% CI 0.79–1.29) for HD, 1.02 (obs = 747, 95% CI 0.80–1.30) for IHD, and 1.08 (obs = 432, 95% CI 0.74–1.59) for AMI. For all three cardiac outcomes, meta-SMRs were heterogeneous, increased with tsfe and tsce and peaked after 25–29 or 10–14 years, respectively. Meta-Cox coefficients showed no association with lugged or unlugged duration of exposure. Some homogeneous but non-significant increase in the hazard ratio (HR) for AMI mortality was found for cumulative exposure (RE-HR=1.10 per 100 mg/m^3^-years; 95% CI 0.92–1.31), but no increase was seen when recent exposure was considered. Inception cohort analyses were not different from the full cohort results. 

## 5. Discussion

### 5.1. Particle Exposures and Cardiovascular Disease Risks

The reported associations between airborne environmental particle exposures and cardiovascular disease and mortality risk have raised public health concern regarding the adequacy of existing environmental regulation of airborne particle pollution. That airborne particles are ubiquitous and that low concentrations have been associated with significant increases in cardiovascular disease and mortality risk in environmental studies underscores the importance of more fully understanding those specific aspects of particle exposures that contribute to disease risk, and subsequently, what can be done to effectively reduce risk and improve public health. For example, 39,054 inhabitants of four Chinese cities were followed for all-cause mortality and for mortality from specific cardiovascular diseases from 1998 to 2009. For each 10 μg/m^3^ increase in PM_10_, the relative risk ratios (RRs) of all-cause, cardiovascular disease, IHD, heart failure, and cerebrovascular disease mortality were 1.24 (95% CI 1.22–1.27), 1.23 (95% CI 1.19–1.26), 1.37 (95% CI 1.28–1.47), 1.11 (95% CI 1.05–1.17), and 1.23 (95% CI1.18–1.28), respectively [4]. These estimates are somewhat larger than the overall effect estimate for cardiovascular mortality (RR=1.11, 95% CI 1.05–1.16 per 10 μg/m^3^) derived by Hoek and colleagues in their review and meta-analysis of studies of long-term air pollution exposure and cardiac and respiratory disease mortality [49]. At the population level, however, even these modest relative risk estimates―presuming that they reflect true causal associations―could represent large numbers of premature deaths.

Complicating the translation of these findings into public health regulations is the fact that airborne particles vary considerably in their physical, chemical and electrostatic properties. They also occur in the environment in a wide range of circumstances, and can change rapidly due to local anthropogenic activity as well as natural forces, such as weather patterns, dust storms and volcanic action. Particles contributing to ambient air pollution vary in size distribution and chemical composition, and are present with other non-particle constituents of air pollution. Among particulate air pollutants are carbon-based compounds, sometimes characterized as “soot” or “black carbon”. As Long *et al.* [50] explained, however, “black carbon” is substantially different from the industrial product “carbon black” and these terms cannot be used interchangeably, nor should these substances be considered similar. Furthermore, risk factors for cardiovascular diseases are numerous, and may play a role in disease development or mortality that reaches back many years or decades before the disease is recognized or cardiovascular death occurs. For these reasons, environmental air pollution studies may be limited in addressing confounding or effect modification by risk factors that vary considerably geographically, over time, and by socioeconomic and underlying community health conditions.

Numerous studies of occupational exposures to airborne particulate matter and risks of cardiovascular disease or mortality have been published. The earliest of these were presented in a narrative review by Sjögren [51], and more recent studies were summarized in a meta-analysis by Fang *et al.* [13]. Fang *et al.* [13] conducted a systematic review of occupational epidemiology studies published between January 1990 and April 2009 on particulate matter (e.g., silica, styrene, diesel exhaust, asphalt fumes, metal or welding fumes) and cardiovascular disease (mortality, morbidity, intermediate cardiovascular endpoints) and identified 697 articles, 37 of which were included (12 mortality, 5 morbidity, 20 intermediate endpoints). Meta-analysis was only used for mortality studies, as other endpoints and study approaches were determined to differ too much. Meta-analysis results for death from IHD (ICD-9 410–414) were SMR = 1.09 (95% CI 0.92–1.30, based on seven studies); and IRR = 1.15 (95% CI 1.06–1.26, based on 4 studies), with the strongest effects seen among workers exposed to crystalline silica. However, the unit of the meta-IRR for IHD mortality is unclear (see Fang *et al.* [13] p. 1784–1785). Several occupational exposures, including CB, titanium dioxide and coal dust―all somewhat more chemically inert than silica—were not among the search terms used, so the direct relevance of the findings to particles of low toxicity is unclear. Further, exposure estimates used in the studies tended to be crude, and the ability to control for smoking and other potential confounders was limited [13].

In addition to the studies included in the meta-analysis by Fang *et al.* [13], we identified the following occupational cohorts of airborne dust-exposed workers, in which cardiovascular risks were reported to be elevated. Toren *et al.* [52] followed a cohort of over 175,000 Swedish construction workers from 1971–2002 and reported a relative risk (RR) of 1.07 (95% CI 1.03–1.12) for airborne exposure to inorganic dusts and IHD. Burstyn *et al.* [53] conducted a mortality study of asphalt workers exposed to asphalt fume and construction dusts and reported SMRs of 1.85 (95% CI 1.17–2.91) for cardiovascular disease and 1.64 for ischemic HD (95% CI 1.13–2.38). A study comparing foundry workers with high to low dust exposures reported SMRs of 1.20 (95% CI 1.04–1.35) for cardiovascular disease and 1.40 (95% CI 1.19–1.74) for IHD [54]. A cohort of 4626 sand workers showed an elevated rate of IHD mortality (SMR 1.22, 95% CI 1.11–1.33) [55]. 

More recent studies include a review of medical insurance claims of nearly 12,000 US aluminum workers, which showed that recent occupational exposure to PM_2.5_ was associated with IHD [56]. In a cohort study of 17,644 German porcelain production workers the authors reported no overall excess mortality for the broad category of diseases of the circulatory system: SMR = 1.00 (95% CI 0.90–1.11) for men, based on 371 observed deaths and SMR = 0.83 (95% CI 0.69–0.98) for women, based on 125 observed deaths. However, restricting analyses to a sub-cohort of Bavarian workers, the SMRs were higher, and for men the SMR was 1.17 (95% CI 1.05–1.31) [57]. 

A cohort of over 74,000 Chinese workers from metal mines and pottery factories was evaluated for mortality risks [58]. A statistically significant elevation was noted for the IHD (SMR = 1.65, 95% CI 1.35–1.99). Pulmonary HD (ICD-10 126–127) generated a HR of 3.44 (95% CI 3.01–3.92) at high levels of cumulative dust exposure. IHD was not elevated at the same level of exposure. High levels were described as more than 4.46 mg/m^3^-years. A study of over 42,000 Chinese workers exposed to crystalline silica included 2846 deaths due to various forms of HD [59]. No increase was noted for myocardial infarction at the highest level of exposure, however, the HR for pulmonary HD (ICD 10–126, 127) was 5.46 (95% CI 4.52–6.61). The authors noted that their results “showed increased pulmonary HD mortality but also among those with low exposure suggesting that silica exposure can increase pulmonary HD mortality.” This finding, however, is limited because of a missing monotonic dose-response: ”Our quantitative exposure–response analyses showed a positive association between silica exposure and IHD mortality among workers with low-level exposure but a negative association among workers with high-level exposure” [59].

A cohort of all manual workers in the Swedish national census of 1980 was identified with information on demographic data and occupation [60]. Exposure to small particles (diameter <1 µm) was associated with AMI with an HR of 1.12 (95% CI 1.09–1.15); the HR for exposure to larger particles was 1.14 (95% CI 1.10–1.18). The authors noted that their explorative study supports the hypothesis that occupational exposure to particles increases the risk of AMI and other IHD. The findings, according to the authors, must be interpreted cautiously due to lack of smoking data.

Among over 265,000 textile workers in Shanghai, no increase in IHD was found; however, mildly elevated risks in ischemic and hemorrhagic stroke were observed [61].

Risk of IHD among a cohort of nearly 9000 US coal miners was increased with increasing levels of exposure [62]. Statistically significant increases and dose-response patterns were noted for IHD at cumulative exposures >64 mg/m^3^-years. Risk was also associated with coal dust rank. The authors were unable to adjust for silica exposure and referred to an earlier study of silica-exposed workers in which an increased risk of IHD was observed [63].

In light of the results of environmental studies, studies of occupational cohorts have shown increased risks of cardiovascular disease, but typically at much higher exposure concentrations. Some authors have proposed that these differences in reported effects may be based on different distributions of particle size [64]. For the sake of clarity we note that dust fractions are defined differently, according to their application: PM_10_ and PM_2.5_ fractions are used in environmental settings (BS EN12341, CFR 40 Part 53) whereas inhalable, thoracic, and alveolar (respirable) fractions are used in occupational settings (ISO 7708, CEN 481). The PM_10_ dust fraction is defined to have a mass sampling distribution with a median effectiveness at an aerodynamic particle diameter of about 10 μm (BS EN12341, CFR 40 Part 53) and it is very similar to the thoracic dust fraction (ISO 7708, CEN 481). The PM_2.5_ dust fraction has no direct counterpart among the dust fractions used in occupational settings. It is similar, however, to the alveolar fraction, although with a tendency to underestimate the latter (BS EN 12341, CFR 40 Part 53, ISO 7708, CEN 481). We note that often misleading definitions of the PM_10_ and PM_2.5_ fractions are presented as if these fractions cover all and only particles with diameters below 10 μm or below 2.5 μm, respectively (e.g., [5,6,65]). Thus, the difference between the dust fractions used in environmental or occupational settings may not be as large as these misleading definitions may indicate. 

On the other hand, some very large recent studies of particulate exposures have not demonstrated clear risks for cardiovascular diseases. For example, within the multicenter European Study of Cohorts for Air Pollution Effects (ESCAPE), long-term exposure to air pollutants and risk of various cardiovascular diseases (CVD) mortality was investigated [66]. The study was based on data from 22 European cohort studies generating a total study population of 367,383, and 9994 CVD deaths including 4992 from IHD, 2264 from MI, and 2484 from cerebrovascular disease. The authors reported, “All hazard ratios were approximately 1.0, except for particle mass and cerebrovascular disease mortality; for PM_2.5_, the hazard ratio was 1.21 (95% CI 0.87–1.69) per 5 μg/m^3^ and for PM_10_, 1.22 (95% CI 0.91–1.63) per 10 μg/m^3^” [66].

Another difference between environmental (general population-based) and occupational (workforce-based) studies may be related to the healthy worker effect, *i.e.*, workers must be reasonably healthy to seek employment and continue employment, whereas the general population includes all individuals, including sick and disabled people [37,67,68]. Population studies of ambient air pollution include susceptible individuals, including individuals whose health is compromised (and who cannot work). The healthy worker effect has been described as being especially pronounced for heart disease mortality [69]. 

### 5.2. Carbon Black Exposure and Cardiovascular Disease Risks

Although today airborne CB dust exposures in the workplace are relatively low and well controlled, historical levels are known to have been quite high, especially in certain production and handling areas (e.g., bag houses prior to automation and enhanced ventilation). Previous evaluations of cardiovascular disease risks among CB manufacturing workers have been limited by methodological shortcomings and incomplete reporting of non-cancer health effects including cardiovascular effects by quantified exposure categories [70,71].

Animal studies have the distinct advantage of being able to control or precisely characterize the composition of the administered exposures, and to observe subtle pathological changes in sacrificed animals. However, while cardiovascular effects can be observed following respiratory exposure to CB, animal studies have not elucidated the mechanism for these effects. One study demonstrated significant inflammatory and other effects in mouse lung and liver following installation of Printex 90, a specific CB product (manufactured and donated by Evonik/Degussa, Frankfurt, Germany) [72]. A further study indicated that the observed cardiovascular effects did not reflect cardiac gene alterations [73]. A number of other studies have also used CB as a model particulate compound to investigate the possible effects of particles on various aspects of cardiovascular function. As example, Jia *et al.* [74], explored the effect on heart rate variability (HRV) by instilling CB into the lungs of male C57BL/6 mice at doses of 0, 0.05, 0.15 and 0.6 mg/kg (each CB dose was instilled three times: on study day 7, 9, and 11). Although slight pulmonary inflammation and myocardial injury were only observed in the 0.6 mg/kg CB exposed group, the authors reported that HRV indices, standard deviation of all normal R–R intervals and the square root of mean of sum of squares of differences between adjacent normal R–R intervals showed significant decreases in the 0.15 and 0.6 mg/kg CB exposed groups. They concluded that CB can disturb cardiac autonomic nervous system function in mice, indicated by the withdrawal of parasympathetic modulation, through mechanisms independent of apparent myocardial and pulmonary injury. Interestingly, they stated, incorrectly, that CB is one of the main components in PM. 

Kim *et al.* [75] intratracheally instilled into rats CB of two primary particle sizes (ultrafine: 28–36 nm and fine: 250–350 nm) at single doses of 1, 3, or 10 mg/kg and sacrificed them at either 24 h or 7 days post-instillation. The authors measured thrombotic activity and determined plasma homocysteine levels, cardiac functionality, and inflammatory responses at these two time periods. The authors found that exposure to the ultrafine CB accelerated platelet-dependent blood clotting at 10 mg/kg, but unexpectedly, both the ultrafine and fine CBs led to prolongation of activated partial thromboplastin time. Both CB particles failed to affect prothrombin time. The fine CB produced a significant elevation in the level of plasma homocysteine. The authors concluded that CB exposure might enhance the cardiovascular risk by inducing hyperhomocysteinemia and platelet hyperactivity, although these effects may be variable depending on particle size and exposure duration. They suggested the homocysteine may thus be a potential biomarker for cardiovascular toxicity following CB exposure.

These studies, and other studies in which rodents receive single or repeated intratracheal instillations of CB, must be interpreted with caution. First, the particle doses are quite massive compared to the “normal” doses that humans might receive through either environmental of occupation exposure. Second―and most importantly―the means of delivering the dose are unphysiological. Intratracheal instillation causes a bolus effect, as often evidenced by acute lung inflammatory effects and thus may elicit corresponding unphysiological responses which may mask or overwhelm subtle true cardiovascular physiological perturbations, possibly related to cardiovascular dysfunction and particle exposure. Ultimately, even where effects can be seen in animal models, problems of extrapolation―across species as well as in terms of exposure levels―are difficult to overcome [76].

In a recent review of human and mouse investigations, Saber *et al.* [77] have proposed that an acute phase pulmonary response may be an underlying mechanistic causal link between particle inhalation and cardiovascular disease. In their review, they present a range of experimental findings in mice that have been exposed to a group of nanoparticles, including carbon black, titanium dioxide and diesel exhaust particles via inhalation or intratracheal instillation. They note the association in mice between particle-induced neutrophil influx and the acute phase pulmonary response and further observe that this neutrophil influx correlates with the well-observed association with the total surface area of the deposited particles. Interestingly, some of these same researchers [78] had previously demonstrated that in mice receiving either carbon black or diesel exhaust particles by nose-only inhalation, the degree of pulmonary inflammation from carbon black exposure was far less that that induced by diesel exhaust particles. Although care must be taken when reading across from experimental results in mice to humans, it may also be worth some caution when reading across the results of this current study of occupational exposure to carbon black to other nanoparticle exposure in other groups of workers and, to air pollution particulates in the general population. 

The current evaluation of cardiovascular disease mortality risks among defined cohorts of industrial CB production workers helps fill a critical void, as it is the first such study to combine quantitative CB exposures with HD, IHD and MI mortality risks. The context for this study―the three established and long-followed cohorts of CB workers in the UK, Germany and the US―presents some distinct advantages in evaluating the risks of these diseases potentially associated with occupational exposure to substantial concentrations of relatively pure CB particles of known particle size distribution and free from other concomitant, including gaseous exposures. The exposures are reasonably well documented and characterized and CB studies may be helpful in pinpointing whether cardiovascular disease risks are associated with occupational CB exposure, and by extension, possibly other particles of low toxicity.

Results from each of the individual cohort studies are presented separately, however, some―especially those from the UK and Germany―were based on relatively small numbers. Therefore, the meta-analysis procedures used in this study to combine cohort-specific results derived according to a common protocol greatly enhanced statistical power, thus improving our ability to identify even small associations. This study essentially included all cohort studies of industrial CB workers published to date, and therefore has the greatest potential for identifying cardiovascular disease mortality risks associated with occupational CB exposure. The availability of reasonably detailed employment histories and exposure assessment efforts in each of the three constituent cohorts allowed quantitative evaluation of risk of cardiovascular mortality by standardized individual CB exposure estimates. Although results did not suggest any, this approach might have allowed identification of exposure thresholds for cardiovascular mortality risks.

Although the individual study findings previously published highlighted some potential differences in mortality patterns (especially with respect to lung cancers) across cohorts, the methods used here largely confirmed that any differences tend to be seen only with respect to “external” referent comparisons (*i.e.,* SMR analyses). These differences are reflected in the heterogeneity measures obtained in the meta-SMR analyses, and were especially strong in the category of all-cause mortality. On the other hand, the Cox analyses compared risks among more highly exposed workers with relatively unexposed workers from the same cohorts (*i.e.,* “internal” comparisons), which avoids some problems associated with comparing disease or mortality risks among workers with those of the general population. 

Intensive epidemiological research into risk factors for heart diseases has identified a number of potential causes of these diseases. Delfino *et al.* [79] summarized many of these, including smoking, LDL cholesterol, hypertension, BMI, diabetes, alcohol intake [80,81,82], genetics [83], social factors [84], diet [85,86,87,88], noise [89,90,91,92], perceived stress, job strain, low decision latitude [93,94,95,96], carbon monoxide [97], styrene [98], ambient temperature [99], and shift-work [100,101,102,103,104]. Some of these factors might be potential confounders in this study. In order to be confounders, these risk factors must also be correlated with exposure. Although we would not expect any to correlate with exposure to CB *per se*, we also lack data to evaluate this. Where we do have some limited data on smoking in the German cohort, it does not appear to have any effect on our results. In the US cohort [15], there was some evidence that the overall prevalence of smoking might be lower than that of the general population, although no individual level data on smoking were available. Ultimately, because we observed no clear or consistent associations between CB exposure indices and risk of any of the cardiovascular disease mortality outcomes, the only concern would be whether a true effect might have been missed because of negative confounding, *i.e.*, the true effect was not seen because those most highly exposed to CB were also more likely to lack other risk factors for the same conditions (*i.e.*, smoking), however, no direct evidence of this is available.

Regardless of the various analytical approaches attempted, results based on meta-analyses of these three cohorts demonstrated no convincing evidence of a relationship between quantitative occupational CB exposure and cardiovascular disease mortality risk―including specific evaluations of all HD, IHD and MI mortality. Analyses included meta-SMRs, which address the research question of whether the occupational cohort exhibits greater mortality rates due to these diseases than expected from an appropriate referent population (generally national or state rates), as well as Cox analyses, which compare exposed workers with the unexposed/least exposed workers. Because observations that “reductions in PM levels are associated with decreases in cardiovascular mortality within a time frame as short as a few years” [5], we additionally “lugged” cumulative exposures which emphasizes more recent exposures in evaluating risks [16,27].

Our results, which contrast with those of many environmental and occupational studies of various particles and mixtures (and in the case of air pollution studies, other toxic substances), suggest that exposure to relatively pure CB may not contribute to increased cardiovascular risk. This again raises the question of whether the chemical composition of particulate pollution might play a greater role in cardiovascular disease development than the quantity or size distribution. Although Landen *et al.* [62] observed increased cardiovascular disease mortality among coal miners, they noted that the size of this effect was smaller than might be predicted based on air pollution studies, and suggested that the difference may be due to the chemical properties of the particles [62]. This might also be the case with CB, which occurs in the workplace as relatively pure carbon, with very low concentrations of metals, PAHs and other contaminants. 

Various mechanisms have been proposed for particle induced cardiovascular effects including systemic inflammation [12,105,106]. Seaton and colleagues published the hypothesis that urban particulate air pollution may provoke alveolar inflammation, with release of mediators capable of increasing blood coagulability in susceptible people [12]. This mechanism would explain the observed increases of cardiovascular deaths associated with episodes of urban pollution. Sjögren [51] expanded this hypothesis to occupational exposure to inhaled particles and to the occurrence of IHD. Long term inhalation of particles from the work environment will hypothetically create a low grade inflammation, which is associated with an increase in plasma fibrinogen. The high concentrations of fibrinogen will then increase the risk of myocardial infarction and IHD.

Brook *et al.* [5] proposed three general intermediary pathways (in their Figure 3): These include pathway 1, the release of proinflammatory mediators (e.g., cytokines, activated immune cells, or platelets) or vasculoactive molecules (e.g., endothelian, possibly histamine, or micro particles) from lung based cells; pathway 2, perturbation of systemic autonomic nerve system balance or heart rhythm by particle interactions with lung receptors or nerves; and pathway 3, potentially the translocation of particulate (*i.e.*, ultrafine particles) or particle constituents (organic compounds, metals) into the systemic circulation. Newby *et al.* [6] described similar potential mechanisms. In their review, Hoek *et al.* [49] identified that in several case–control studies drawing cases from registries or clinical review associations are seen between NO_2_ exposure and fatal MI; similar results were not observed with non-fatal MI. This suggests that air pollution might particularly aggravate disease progression caused by other factors leading to mortality in those most compromised [49].

Inflammatory markers have been increased in relation to particle exposure as manifested by elevations in Interleukin (IL)-6 [107]. Miller *et al.* [106] have proposed “that alveolar inflammation provoked by ultra-fine particles, in addition to promoting exacerbations of lung disease has an additional effect on the coagulability of blood, increasing the susceptibility of individuals to acute episodes of cardiovascular disease.” They further proposed that it was the “number, composition and size of the particle of most importance and not the mass of particles” [106], which according to Seaton *et al.* accounts for why excess deaths occur in people indoors and why “similar serious effects are not seen in industrial workers exposed to much higher concentrations of dust measured by weight” [12]. 

In a study of 81 CB workers in China, a statistically significant association was shown between exposure to CB and serum levels of the inflammatory mediators (IL-1β, IL-6, IL-8, and TNFα). Multiple logistic regression analyses showed an association between CB exposure and increases in cytokines after adjusting for age, BMI, smoking, and drinking habits. The authors noted that their study was the “first to provide persuasive evidence for CB-induced pro inflammatory cytokines in human beings” [108]. Similar results were observed in mice: levels of IL-6 and IL-8 significantly increased in the 7 day and 14 day CB exposure groups compared with the control groups. These results suggest that higher doses of exposure can be associated with inflammation and manifested as inflammatory mediators in the serum. The results, however, need to be evaluated in the context of the level of exposure in the Zhang *et al.* study [108]. Average worker exposure, measured as inhalable dust concentration, was 14.9 mg/m^3^, in comparison to US guidelines of 3.5 mg/m^3^ (total) and far lower carbon black dust concentrations reported in US and European production plants [76]. Thus, whether similar inflammatory marker effects would be noted at lower levels of exposure is unclear.

Based on the evolving body of evidence regarding inhalable particles and cardiovascular disease risks, including the primary findings of this study, more attention should be paid to the combination of physical and chemical properties of particulate air pollutants, as well as the context in which people are exposed. Our results suggest that occupational exposure to substantial airborne concentrations of commercially produced pure CB particles is not associated with increased risk of cardiovascular disease, IHD or MI mortality. Because the physical-chemical characteristics of occupational exposure to carbon black may be different from that of other particulate exposures (e.g., ambient black carbon, see Sjögren *et al.* [50]), these results may not be relevant for other exposure scenarios, including mixtures of particulates and gaseous constituents, even at lower concentrations.

## 6. Conclusions

Epidemiological studies have demonstrated associations between environmental particle exposure and cardiac disease and mortality; however, few have examined such effects from pure carbon such as manufactured CB particles. 

We combined SMR and Cox proportional hazards results from cohort studies of US, UK and German CB production workers, particularly for IHD and AMI. Results from each of the individual cohort studies are presented separately, some―especially those from the UK and Germany―were based on relatively small numbers. Therefore, the meta-analysis procedures used in this study to combine cohort-specific results derived according to a common protocol greatly enhanced statistical power, thus improving our ability to identify even small associations. This study essentially included all cohort studies of industrial CB workers published to date, and therefore has the greatest potential for identifying cardiovascular disease mortality risks associated with occupational CB exposure. The availability of reasonably detailed employment histories and exposure assessment efforts in each of the three constituent cohorts allowed quantitative evaluation of risk of cardiovascular mortality by standardized individual CB exposure estimates. Although results did not suggest any, this approach might have allowed identification of exposure thresholds for cardiovascular mortality risks.

Meta-SMRs were unexceptional. Meta-Cox coefficients showed no association with lugged or unlugged duration of exposure. Our results do not demonstrate that CB exposure increases cardiac disease mortality. Based on the evolving body of evidence regarding inhalable particles and cardiovascular disease risks, more attention should be paid to the combination of physical and chemical properties of particulate air pollutants, as well as the context in which people are exposed. 

## Figures and Tables

**Table 1:** Observed (Obs) deaths, standardised mortality ratios (SMRs), and 95% confidence intervals (CI) for overall ^a^ and cardiac ^b^ mortality based on national rates by period from hire, in carbon black production workers (full cohorts).

Period from Hire ^c^ (Year)	US			UK			Germany		
	Obs	SMR	(95% CI)	Obs	SMR	(95% CI)	Obs	SMR	(95% CI)
***Overall*^a^**									
0–4	27	0.49	(0.33–0.72)	11	1.05	(0.52–1.88)	4	1.13	(0.31–2.89)
5–9	50	0.57	(0.43–0.76)	18	0.93	(0.55–1.46)	6	0.64	(0.23–1.39)
10–14	65	0.55	(0.43–0.71)	31	1.03	(0.70–1.46)	22	1.20	(0.75–1.82)
15–19	117	0.74	(0.61–0.89)	51	1.11	(0.83–1.46)	42	1.33	(0.96–1.79)
20–24	139	0.69	(0.58–0.81)	74	1.20	(0.95–1.51)	59	1.35	(1.03–1.75)
25–29	191	0.78	(0.68–0.90)	83	1.10	(0.87–1.36)	77	1.62	(1.28–2.03)
≥30	1358	0.93	(0.88–0.98)	309	1.13	(1.01–1.26)	122	1.06	(0.88–1.27)
Total	1947	0.84	(0.80–0.88)	577	1.12	(1.03–1.21)	332	1.24	(1.11–1.37)
***Heart Disease*^b^**									
0–4	2	0.24	(0.03–0.85)	1	0.33	(0.01–1.83)	1	2.42	(0.06–13.48)
5–9	13	0.72	(0.38–1.23)	4	0.61	(0.17–1.56)	0	0.00	(0.00–2.24)
10–14	17	0.56	(0.33–0.90)	13	1.19	(0.63–2.03)	7	1.54	(0.62–3.18)
15–19	33	0.70	(0.48–0.98)	19	1.11	(0.67–1.73)	8	0.91	(0.39–1.79)
20–24	41	0.62	(0.45–0.85)	23	1.01	(0.64–1.52)	18	1.38	(0.82–2.18)
25–29	58	0.71	(0.54–0.91)	34	1.27	(0.88–1.77)	31	2.14	(1.46–3.04)
≥30	433	0.93	(0.84–1.02)	75	1.03	(0.81–1.29)	38	1.03	(0.73–1.41)
Total	597	0.83	(0.76–0.90)	169	1.00	(0.85–1.16)	103	1.29	(1.05–1.56)

^a^ ICD-9 001–999; ^b^ ICD-9 410–429, (US: ICD9 410–414, 420–429); ^c^ period from hire = time since first exposure (tsfe).

**Table 2:** Meta analyses of standardised mortality ratios (SMRs) for overall ^a^ and cardiac ^b^ mortality based on national rates by period from hire, in carbon black production workers of three full cohorts combined (US, UK, Germany).

Period from Hire ^c^ (Year)	Obs	Fixed Effects			Random Effects			Heterogeneity		
		SMR	(95% CI)	*p*_eff_^d^	SMR	(95% CI)	*p*_eff_^d^	I^2 e^	chi^2^_het_^f^	*p*_het_^f^
***Overall*^a^**										
0–4	42	0.64	(0.46–0.88)	0.01	0.75	(0.41–1.37)	0.35	59.9	4.99	0.08
5–9	74	0.65	(0.51–0.82)	<0.01	0.67	(0.49–0.92)	0.01	26.9	2.73	0.26
10–14	118	0.75	(0.62–0.90)	<0.01	0.86	(0.52–1.43)	0.57	84.4	12.82	<0.01
15–19	210	0.92	(0.80–1.05)	0.22	1.01	(0.70–1.46)	0.95	83.7	12.28	<0.01
20–24	272	0.92	(0.82–1.04)	0.19	1.03	(0.66–1.61)	0.90	91.9	24.84	<0.01
25–29	351	0.99	(0.89–1.10)	0.86	1.11	(0.72–1.70)	0.64	93.0	28.61	<0.01
≥30	1789	0.97	(0.93–1.02)	0.20	1.03	(0.89–1.18)	0.71	80.5	10.24	0.01
Total	2856	0.94	(0.90–0.98)	0.00	1.05	(0.81–1.34)	0.73	96.9	65.18	<0.01
***Heart Disease*^b^**										
0–4	4	0.42	(0.12–1.50)	0.18	0.43	(0.12–1.59)	0.21	4.4	2.09	0.35
5–9	17	0.65	(0.39–1.10)	0.11	0.40	(0.08–1.94)	0.25	74.8	7.94	0.02
10–14	37	0.88	(0.62–1.24)	0.45	0.96	(0.52–1.77)	0.89	65.8	5.85	0.05
15–19	60	0.83	(0.64–1.09)	0.18	0.85	(0.63–1.14)	0.28	16.1	2.38	0.30
20–24	82	0.84	(0.67–1.06)	0.14	0.93	(0.58–1.49)	0.75	75.1	8.02	0.02
25–29	123	1.10	(0.91–1.32)	0.33	1.23	(0.64–2.36)	0.53	91.6	23.93	<0.01
≥30	546	0.95	(0.87–1.03)	0.21	0.95	(0.87–1.03)	0.21	0.0	0.91	0.63
Total	869	0.91	(0.85–0.97)	<0.01	1.01	(0.79–1.29)	0.95	89.0	18.10	<0.01

^a^ ICD-9 001–999; ^b^ ICD-9 410–429, (US: ICD9 410–414, 420–429); ^c^ period from hire = time since first exposure (tsfe); ^d^ *p-*value of effect; ^e^ proportion of variability due to heterogeneity (%); ^f^ heterogeneity chi^2^ and heterogeneity *p*-value.

**Table 3:** Standardised mortality ratios (SMRs) based on national rates for cardiac ^a,b^ mortality by period from hire, in carbon black production workers (full cohorts).

Period from Hire ^c^ (Year)	US			UK			Germany		
	Obs	SMR	(95% CI)	Obs	SMR	(95% CI)	Obs	SMR	(95% CI)
***Ischaemic Heart Disease*^a^**									
0–4	1	0.14	(0.00–0.80)	1	0.38	(0.01–2.12)	1	3.79	(0.10–21.12)
5–9	13	0.86	(0.46–1.46)	4	0.67	(0.18–1.71)	0	0.00	(0.00–3.26)
10–14	15	0.58	(0.32–0.95)	13	1.27	(0.68–2.17)	5	1.50	(0.49–3.51)
15–19	31	0.75	(0.51–1.06)	18	1.12	(0.66–1.77)	7	1.08	(0.43–2.23)
20–24	34	0.59	(0.41–0.82)	21	0.99	(0.61–1.51)	10	1.05	(0.50–1.93)
25–29	50	0.69	(0.51–0.91)	33	1.34	(0.92–1.88)	23	2.18	(1.38–3.26)
≥30	367	0.94	(0.84–1.04)	71	0.96	(0.75–1.11)	29	1.09	(0.73–1.57)
Total	511	0.84	(0.76–0.91)	161	1.04	(0.89–1.22)	75	1.30	(1.02–1.62)
***Acute Myocardial Infarction*^b^**									
0–4	1	0.21	(0.01–1.14)	1	0.43	(0.01–2.37)	1	4.50	(0.11–25.08)
5–9	9	0.86	(0.39–1.64)	4	0.81	(0.22–2.07)	0	0.00	(0.00–3.98)
10–14	6	0.34	(0.12–0.74)	12	1.50	(0.78–2.63)	5	1.88	(0.61–4.39)
15–19	16	0.59	(0.33–0.95)	15	1.26	(0.70–2.07)	6	1.21	(0.44–2.62)
20–24	23	0.61	(0.39–0.92)	17	1.15	(0.67–1.84)	7	0.99	(0.40–2.05)
25–29	32	0.72	(0.49–1.02)	26	1.63	(1.07–2.39)	19	2.57	(1.55–4.01)
≥30	177	0.92	(0.79–1.06)	30	0.76	(0.51–1.09)	25	1.45	(0.94–2.14)
Total	264	0.79	(0.70–0.89)	105	1.08	(0.88–1.31)	63	1.56	(1.20–1.99)

^a^ ICD-9 410–414 (US: ICD-9 410–414, 429.2, ICD-10 I20–I22, I24–I25, I51.3, I51.6); ^b^ ICD-9 410, ICD-10 I21; ^c^ period from hire = time since first exposure (tsfe).

**Table 4:** Meta analyses of standardised mortality ratios (SMRs) for cardiac ^a,b^ mortality based on national rates by period from hire, in carbon black production workers of three full cohorts combined (US, UK, Germany).

Period from Hire ^c^ (Year)	Obs	Fixed Effects			Random Effects			Heterogeneity		
		SMR	(95% CI)	*p*_eff_^d^	SMR	(95% CI)	*p*_eff_^d^	I^2 e^	chi^2^_het_^f^	*p*_het_^f^
***Ischaemic Heart Disease*^a^**										
0–4	3	0.60	(0.13–2.83)	0.52	0.60	(0.09–4.01)	0.59	33.6	3.01	0.22
5–9	17	0.77	(0.46–1.28)	0.31	0.47	(0.10–2.25)	0.34	74.4	7.83	0.02
10–14	33	0.90	(0.62–1.30)	0.58	0.97	(0.52–1.79)	0.91	60.2	5.02	0.08
15–19	56	0.89	(0.67–1.17)	0.40	0.89	(0.67–1.17)	0.40	0.0	1.89	0.39
20–24	65	0.76	(0.58–0.98)	0.03	0.80	(0.54–1.20)	0.28	53.5	4.30	0.12
25–29	106	1.08	(0.89–1.32)	0.43	1.24	(0.64–2.41)	0.52	90.4	20.81	<0.01
≥30	467	0.95	(0.87–1.04)	0.27	0.95	(0.87–1.04)	0.27	0.0	0.58	0.75
Total	747	0.92	(0.85–0.98)	0.02	1.02	(0.80–1.30)	0.86	87.0	15.35	<0.01
***Acute Myocardial Infarction*^b^**										
0–4	3	0.73	(0.15–3.47)	0.69	0.73	(0.12–4.51)	0.73	26.7	2.73	0.26
5–9	13	0.78	(0.43–1.42)	0.42	0.50	(0.10–2.53)	0.40	73.3	7.50	0.02
10–14	23	1.08	(0.69–1.69)	0.73	1.00	(0.37–2.70)	0.99	77.5	8.87	0.01
15–19	37	0.89	(0.63–1.26)	0.52	0.92	(0.54–1.59)	0.78	55.3	4.47	0.11
20–24	47	0.82	(0.61–1.12)	0.21	0.85	(0.55–1.32)	0.48	45.7	3.68	0.16
25–29	77	1.29	(1.02–1.63)	0.03	1.43	(0.68–2.99)	0.35	89.7	19.36	<0.01
≥30	232	0.94	(0.82–1.07)	0.35	0.98	(0.73–1.32)	0.89	64.3	5.60	0.06
Total	432	0.94	(0.85–1.03)	0.18	1.08	(0.74–1.59)	0.68	92.1	25.31	<0.01

^a^ ICD-9 410–414 (US: ICD-9 410–414, 429.2, ICD-10 I20–I22, I24–I25, I51.3, I51.6); ^b^ ICD-9 410, ICD-10 I21; ^c^ period from hire = time since first exposure (tsfe); ^d^ *p*-value of effect; ^e^ proportion of variability due to heterogeneity (%); ^f^ heterogeneity chi^2^ and heterogeneity *p*-value.

**Table 5:** Standardised mortality ratios (SMRs) for overall ^a^ and cardiac ^b^ mortality based on national rates by period from leaving employment, in carbon black production workers (inception cohorts, US: follow-up ≥ 1979).

Period from Leaving Employment ^c^ (Year)	US			UK			Germany		
	Obs	SMR	(95% CI)	Obs	SMR	(95% CI)	Obs	SMR	(95% CI)
***Overall*^a^**									
<1 ^d^	46	0.40	(0.29–0.53)	33	1.15	(0.79–1.61)	35	0.76	(0.53–1.06)
1–4	38	0.82	(0.58–1.13)	19	1.00	(0.60–1.56)	23	1.34	(0.85–2.02)
5–9	43	0.75	(0.54–1.01)	31	0.98	(0.66–1.38)	33	1.45	(1.00–2.03)
10–14	52	0.92	(0.68–1.20)	50	1.22	(0.91–1.61)	43	2.00	(1.45–2.70)
15–19	67	1.11	(0.86–1.41)	52	1.15	(0.86–1.50)	37	2.14	(1.50–2.95)
20–24	55	0.86	(0.65–1.12)	50	1.08	(0.80–1.42)	27	1.90	(1.25–2.77)
≥25	581	1.07	(0.98–1.16)	175	1.08	(0.92–1.25)	20	1.19	(0.72–1.83)
Total	882	0.93	(0.87–1.00)	410	1.09	(0.99–1.21)	218	1.40	(1.22–1.60)
***Heart Disease*^b^**									
<1 ^d^	12	0.47	(0.24–0.83)	10	1.00	(0.48–1.84)	8	0.77	(0.33–1.52)
1–4	11	0.97	(0.48–1.73)	6	0.91	(0.34–1.99)	6	1.18	(0.43–2.56)
5–9	15	1.01	(0.57–1.67)	14	1.26	(0.69–2.11)	12	1.73	(0.89–3.02)
10–14	15	0.98	(0.55–1.61)	15	1.06	(0.59–1.75)	15	2.31	(1.29–3.81)
15–19	14	0.82	(0.45–1.38)	14	0.90	(0.49–1.50)	6	1.18	(0.43–2.58)
20–24	13	0.68	(0.36–1.16)	15	0.96	(0.54–1.58)	11	2.65	(1.32–4.73)
≥25	181	1.06	(0.91–1.23)	53	1.08	(0.81–1.42)	2	0.40	(0.05–1.45)
Total	261	0.95	(0.84–1.08)	127	1.04	(0.87–1.24)	60	1.39	(1.06–1.79)

^a^ ICD-9 001–999; ^b^ ICD-9 410–429, (US: ICD-9 410–414, 420–429); ^c^ period from leaving employment = time since cessation of exposure (tsce); ^d^ Includes in-service deaths.

**Table 6:** Meta analyses of standardised mortality ratios (SMRs) for overall ^a^ and cardiac ^b^ mortality based on national rates by period from leaving employment, in carbon black production workers of three inception cohorts combined (US, UK, Germany).

Period from Leaving Employment ^c^ (Year)	Obs	Fixed Effects			Random Effects			Heterogeneity		
		SMR	(95% CI)	*p*_eff_^d^	SMR	(95% CI)	*p*_eff_^d^	I^2 e^	chi^2^_het_^f^	*p*_het_^g^
***Overall*^a^**										
<1 ^f^	114	0.66	(0.54–0.79)	<0.01	0.70	(0.37–1.31)	0.26	90.6	21.17	<0.01
1–4	80	0.99	(0.79–1.24)	0.90	1.01	(0.75–1.35)	0.96	36.0	3.12	0.21
5–9	107	0.99	(0.81–1.21)	0.92	1.01	(0.69–1.49)	0.96	73.7	7.59	0.02
10–14	145	1.27	(1.08–1.51)	0.01	1.30	(0.84–2.02)	0.24	85.2	13.50	<0.01
15–19	156	1.31	(1.11–1.54)	<0.01	1.38	(0.94–2.02)	0.10	81.2	10.63	0.01
20–24	132	1.10	(0.92–1.31)	0.30	1.18	(0.78–1.79)	0.43	80.8	10.41	0.01
≥25	776	1.07	(1.00–1.15)	0.06	1.07	(1.00–1.15)	0.06	0.0	0.20	0.90
Total	1510	1.03	(0.98–1.09)	0.24	1.12	(0.90–1.39)	0.31	93.3	29.74	<0.01
***Heart Disease*^b^**										
<1 ^g^	30	0.69	(0.47–1.02)	0.06	0.70	(0.44–1.10)	0.12	26.3	2.71	0.26
1–4	23	1.00	(0.64–1.57)	1.00	1.00	(0.64–1.57)	1.00	0.0	0.18	0.91
5–9	41	1.27	(0.92–1.77)	0.15	1.27	(0.92–1.77)	0.15	0.0	1.65	0.44
10–14	45	1.34	(0.98–1.83)	0.07	1.34	(0.78–2.29)	0.29	66.4	5.95	0.05
15–19	34	0.91	(0.63–1.30)	0.59	0.91	(0.63–1.30)	0.59	0.0	0.46	0.80
20–24	39	1.13	(0.81–1.59)	0.47	1.18	(0.55–2.53)	0.66	80.2	10.10	0.01
≥25	236	1.06	(0.93–1.21)	0.38	1.06	(0.93–1.21)	0.38	0.0	1.29	0.53
Total	448	1.03	(0.93–1.13)	0.59	1.08	(0.89–1.31)	0.42	69.6	6.57	0.04

^a^ ICD-9 001–999; ^b^ ICD-9 410–429, (US: ICD-9 410–414, 420–429); ^c^ period from leaving employment= time since cessation of exposure (tsce); ^d^ *p*-value of effect; ^e^ proportion of variability due to heterogeneity (%); ^f^ heterogeneity chi^2^ and heterogeneity *p*-value; ^g^ includes in-service deaths.

**Table 7:** Standardised mortality ratios (SMRs) for cardiac ^a,b^ mortality based on national rates by period from leaving employment, in carbon black production workers (inception cohorts, US: follow-up ≥ 1979).

Period from Leaving Employment ^c^ (Year)	US			UK			Germany		
	Obs	SMR	(95% CI)	Obs	SMR	(95% CI)	Obs	SMR	(95% CI)
***Ischaemic Heart Disease*^a^**									
<1 ^d^	10	0.49	(0.24–0.91)	10	1.09	(0.52–2.00)	7	0.95	(0.38–1.95)
1–4	9	0.97	(0.44–1.84)	6	1.00	(0.37–2.17)	5	1.31	(0.42–3.04)
5–9	12	0.99	(0.51–1.73)	14	1.36	(0.74–2.28)	7	1.36	(0.55–2.79)
10–14	14	1.11	(0.60–1.86)	15	1.15	(0.64–1.90)	12	2.49	(1.29–4.35)
15–19	12	0.86	(0.44–1.50)	13	0.90	(0.48–1.55)	2	0.55	(0.07–1.97)
20–24	11	0.69	(0.34–1.23)	13	0.91	(0.48–1.55)	9	3.00	(1.37–5.69)
≥25	146	1.03	(0.87–1.21)	50	1.14	(0.85–1.51)	1	0.27	(0.01–1.53)
Total	214	0.95	(0.82–1.08)	121	1.09	(0.91–1.30)	43	1.36	(0.99–1.84)
***Acute Myocardial Infarction*^c^**									
<1 ^d^	8	0.68	(0.29–1.34)	10	1.38	(0.66–2.54)	6	1.02	(0.37–2.21)
1–4	5	0.99	(0.32–2.30)	5	1.10	(0.36–2.58)	5	1.77	(0.58–4.14)
5–9	6	0.97	(0.35–2.11)	10	1.36	(0.65–2.51)	5	1.36	(0.44–3.18)
10–14	7	1.06	(0.43–2.19)	11	1.25	(0.62–2.23)	10	3.00	(1.44–5.52)
15–19	7	1.00	(0.40–2.06)	12	1.28	(0.66–2.24)	2	0.78	(0.09–2.82)
20–24	7	0.83	(0.33–1.72)	11	1.24	(0.62–2.21)	7	3.33	(1.34–6.87)
≥25	62	0.91	(0.70–1.17)	27	1.16	(0.77–1.69)	1	0.42	(0.01–2.32)
Total	102	0.90	(0.73–1.09)	86	1.24	(0.99–1.53)	36	1.58	(1.11–2.19)

^a^ ICD-9 410–414; ^b^ ICD-9 410; ^c^ period from leaving employment = time since cessation of exposure (tsce); ^d^ Includes in-service deaths.

**Table 8:** Meta analyses of standardised mortality ratios (SMRs) for cardiac ^a,b^ mortality based on national rates by period from leaving employment, in carbon black production workers of three inception cohorts combined (US: follow-up ≥ 1979, UK, Germany).

Period from Leaving Employment ^c^ (Year)	Obs	Fixed Effects			Random Effects			Heterogeneity		
		SMR	(95% CI)	*p*_eff_^d^	SMR	(95% CI)	*p*_eff_^d^	I^2 e^	chi^2^_het_^f^	*p*_het_^f^
***Ischaemic Heart Disease*^a^**										
<1 ^g^	27	0.78	(0.52–1.18)	0.24	0.79	(0.48–1.30)	0.35	32.1	2.94	0.23
1–4	20	1.05	(0.65–1.71)	0.84	1.05	(0.65–1.71)	0.84	0.0	0.25	0.88
5–9	33	1.21	(0.84–1.75)	0.31	1.21	(0.84–1.75)	0.31	0.0	0.67	0.72
10–14	41	1.42	(1.02–1.97)	0.04	1.45	(0.88–2.38)	0.15	56.7	4.62	0.10
15–19	27	0.86	(0.57–1.29)	0.46	0.86	(0.57–1.29)	0.46	0.0	0.32	0.85
20–24	33	1.14	(0.79–1.65)	0.49	1.21	(0.53–2.79)	0.65	80.1	10.05	0.01
≥25	197	1.05	(0.91–1.22)	0.47	1.05	(0.91–1.22)	0.47	0.0	1.34	0.51
Total	378	1.03	(0.93–1.14)	0.55	1.08	(0.90–1.29)	0.42	59.8	4.98	0.08
***Acute Myocardial Infarction*^b^**										
<1 ^g^	24	1.01	(0.65–1.57)	0.96	1.01	(0.65–1.57)	0.96	0.0	1.87	0.39
1–4	15	1.25	(0.71–2.20)	0.45	1.25	(0.71–2.20)	0.45	0.0	0.77	0.68
5–9	21	1.24	(0.77–1.99)	0.37	1.24	(0.77–1.99)	0.37	0.0	0.41	0.82
10–14	28	1.65	(1.10–2.46)	0.02	1.62	(0.85–3.05)	0.14	59.0	4.88	0.09
15–19	21	1.14	(0.71–1.82)	0.59	1.14	(0.71–1.82)	0.59	0.0	0.42	0.81
20–24	25	1.46	(0.95–2.24)	0.09	1.49	(0.70–3.17)	0.30	66.7	6.00	0.05
≥25	90	0.97	(0.79–1.21)	0.81	0.97	(0.79–1.21)	0.81	0.0	1.43	0.49
Total	224	1.11	(0.97–1.27)	0.12	1.18	(0.87–1.60)	0.28	78.6	9.35	0.01

^a^ ICD-9 410–414 (US: ICD-9 410–414, 429.2, ICD-10 I20–I22, I24–I25, I51.3, I51.6); ^b^ ICD-9 410, ICD-10 I21; ^c^ period from leaving employment = time since cessation of exposure (tsce); ^d^ *p*-value of effect; ^e^ proportion of variability due to heterogeneity (%); ^f^ heterogeneity chi^2^ and heterogeneity *p*-value; ^g^ includes in-service deaths.

**Table 9:** Meta relative risk estimates and meta *Z*-statistics for acute myocardial infarction ^a^ obtained from Cox regression (internal analysis) and based on continuous measures of duration of employment in three full cohorts combined (USA, UK, Germany; 432 observed cases).

Duration of Employment ^b^	Relative Risk ^c^	(95% CI)	chi^2^_het_^d^	*p*_het_^d^	Relative Risk ^e^	(95% CI)	chi^2^_het_^d^	*p*_het_^d^
***Fixed Effects***								
Cumulative employment	1.00	(0.92–1.10)	0.80	0.67	1.01	(0.92–1.11)	0.23	0.89
Recent empl. (lug = 5)	0.59	(0.30–1.14)	0.05	0.98	0.61	(0.31–1.20)	0.05	0.97
Recent empl. (lug = 10)	0.83	(0.60, 1.14)	0.33	0.85	0.85	(0.61–1.19)	0.34	0.85
***Random Effects***								
Cumulative emplyoment	1.00	(0.92–1.10)	0.80	0.67	1.01	(0.92–1.11)	0.23	0.89
Recent empl. (lug = 5)	0.59	(0.30–1.14)	0.05	0.98	0.61	(0.31–1.20)	0.05	0.97
Recent empl. (lug = 10)	0.83	(0.60–1.14)	0.33	0.85	0.85	(0.61–1.19)	0.34	0.85
***Meta-Z, Fixed Effects***								
	**Z ^f^**	***p*^f^**	**chi^2^_het_^d^**	***p*_het_^d^**	**Z ^f^**	***p*^f^**	**chi^2^_het_^d^**	***p*_het_^d^**
Cumulative employment	0.12	0.87	0.92	0.63	0.21	0.78	0.36	0.83
Recent empl.(lug = 5)	−1.03	0.16	0.12	0.94	−0.92	0.21	0.08	0.96
Recent empl. (lug = 10)	−0.95	0.20	1.28	0.53	−0.81	0.28	1.15	0.56

^a^ ICD-9 410, ICD-10 I21; ^b^ Units of 10y; ^c^ Age is basic time variable with additional adjustment for year of birth (continuous variable); ^d^ heterogeneity chi^2^ and heterogeneity *p*-value; ^e^ Additional adjustment for plant group (company) in US, plant group in UK, one plant in Germany (no further adjustment); ^f^ weighted by number of expected cases based on national rates (US: 335.5, UK: 97.4, Germany: 40.4) and *p*-value of effect.

**Table 10:** Risk coefficients for acute myocardial infarction ^a^ obtained from Cox regression (internal analysis) and based on categories of estimated carbon black exposure in three full cohorts (US, UK, Germany; 432 observed cases).

Exposure Metric ^b^	(*n*)	Risk Coefficient ^c^	(95% CI)	*Z*-Value ^d^	AIC	Risk Coefficient ^e^	(95% CI)	*Z*-Value ^d^	AIC
***US*^f^**									
Cumulative exposure									
<20	43	(ref)			4043.04	(ref)			4030.37
20–<50	51	0.30	(−0.10–0.71)	1.47		0.35	(−0.06–0.76)	1.68	
50–<100	46	0.19	(−0.23–0.61)	0.90		0.28	(−0.15–0.70)	1.27	
≥100	124	0.49	(0.14–0.85)	2.74		0.53	(0.17–0.89)	2.86	
Recent exposure (lug=10)									
<5	206	(ref)			4050.16	(ref)			4037.55
5–<10	9	−0.18	(−0.85–0.50)	−0.51		−0.09	(−0.77–0.59)	−0.27	
10–<20	12	−0.09	(−0.68–0.51)	−0.29		0.06	(−0.54–0.66)	0.19	
≥20	37	0.21	(−0.17–0.60)	1.08		0.26	(−0.13–0.65)	1.32	
*UK*									
Cumulative exposure									
<20	33	(ref)			1355.11	(ref)			1354.37
20–<50	30	0.13	(−0.37–0.62)	0.50		0.20	(−0.30–0.70)	0.77	
50–<100	15	−0.18	(−0.80–0.43)	−0.59		−0.07	(−0.69–0.56)	−0.21	
≥100	27	0.11	(−0.41–0.63)	0.41		0.29	(−0.26–0.85)	1.03	
Recent exposure (lug=10)									
<5	76	(ref)			1353.49	(ref)			1353.5
5–<10	2	−1.02	(−2.44–0.40)	−1.41		−0.95	(−2.38–0.47)	−1.31	
10–<20	6	−0.15	(−1.01–0.72)	−0.33		−0.03	(−0.91–0.85)	−0.07	
≥20	21	−0.03	(−0.60–0.54)	−0.11		0.09	(−0.49–0.67)	0.30	
*Germany*									
Cumulative exposure									
<20	19	(ref)			701.58	(ref)			482.82
20–<50	24	0.50	(−0.11–1.11)	1.62		0.12	(−0.60–0.83)	0.32	
50–<100	15	0.25	(−0.45–0.95)	0.69		−0.21	(−1.05–0.64)	−0.48	
≥100	5	0.09	(0.93–1.11)	0.17		−0.07	(−1.22–1.08)	−0.13	
Recent exposure (lug=10)									
<5	47	(ref)			698.99	(ref)			475.93
5–<10	3	−0.61	(−1.81–0.59)	−0.99		−1.20	(−2.67–0.27)	−1.59	
10–<20	11	0.48	(−0.26–1.22)	1.27		0.14	(−0.72–0.99)	0.31	
≥20	2	−0.84	(−2.33–0.64)	−1.11		−1.62	(−3.70–0.45)	−1.54	

^a^ ICD-9 410, ICD-10 I21; ^b^ Units of 100 mg·m^−3^-years for UK and US and 100 unit-years for Germany; ^c^ Age is basic time variable with additional adjustment for year of birth (categorized in decades); ^d^ Risk coefficient/standard error of risk coefficient; ^e^ Additional adjustment for plant group (company) in US, plant group in UK, smoking status in Germany (one plant only); ^f^ Full cohort (*n* = 6634).

**Table 11:** Meta relative risk estimates and meta *Z*-statistics for acute myocardial infarction ^a^ obtained from Cox regression (internal analysis) and based on continuous measures of estimated carbon black exposure in three full cohorts combined (US, UK, Germany; 432 observed cases).

Exposure Metric ^b^	Relative Risk ^c^	(95% CI)	chi^2^_het_^d^	*p*_het_^d^	Relative Risk ^e^	(95% CI)	chi^2^_het_^d^	*p*_het_^d^
***Random Effects***								
Cumulative exposure	1.05	(0.99–1.10)	0.39	0.82	1.10	(0.92–1.31)	3.56	0.17
Recent exp. (lug = 5)	0.95	(0.54–1.67)	1.16	0.56	0.88	(0.51–1.53)	0.93	0.63
Recent exp. (lug = 10)	0.96	(0.73–1.27)	0.46	0.79	0.93	(0.70–1.22)	0.23	0.89
***Meta-Z, Fixed Effects***								
	**Z ^f^**	***p*^f^**	**chi^2^_het_^d^**	***p*_het_^d^**	**Z ^f^**	***p*^f^**	**chi^2^_het_^d^**	***p*_het_^d^**
Cumulative exposure	1.25	0.09	4.36	0.11	1.19	0.11	2.75	0.25
Recent exp.(lug = 5)	−0.13	0.86	0.89	0.64	−0.34	0.65	0.40	0.82
Recent exp. (lug = 10)	−0.17	0.82	0.36	0.83	−0.38	0.61	0.21	0.90

^a^ ICD-9 410, ICD-10 I21; ^b^ Units of 100 mg·m^−3^-years for US and UK and 100 unit-years for Germany; ^c^ Age is basic time variable with additional adjustment for year of birth (continuous variable); ^d^ heterogeneity chi^2^ and heterogeneity *p*-value; ^e^ Additional adjustment for plant group (company) in USA, plant group in UK, one plant in Germany (no further adjustment); ^f^ weighted by number of expected cases based on national rates (USA: 335.5, UK: 97.4, Germany: 40.4) and *p*-value of effect.

**Table 12:** Meta relative risk estimates and meta *Z*-statistics for acute myocardial infarction ^a^ obtained from Cox regression (internal analysis) and based on continuous measures of estimated carbon black exposure in two inception cohorts combined (US: follow-up ≥ 1979, Germany; 138 observed cases).

Exposure Metric ^b^	Relative Risk ^c^	(95% CI)	chi^2^_het_^d^	*p*_het_^d^	Relative Risk ^e^	(95% CI)	chi^2^_het_^d^	*p*_het_^d^
***Random Effects***								
Cumulative exposure	1.08	(1.00–1.16)	0.80	0.37	1.05	(0.97–1.13)	0.88	0.35
Recent exp. (lug = 5)	1.32	(0.22–7.84)	0.09	0.77	0.99	(0.16–6.23)	0.12	0.73
Recent exp. (lug = 10)	1.10	(0.49–2.45)	0.29	0.59	0.94	(0.41–2.19)	0.36	0.55
***Meta-Z, Fixed Effects***								
	**Z ^f^**	***p*^f^**	**chi^2^_het_^d^**	***p*_het_^d^**	**Z ^f^**	***p*^f^**	**chi^2^_het_^d^**	***p*_het_^d^**
Cumulative exposure	1.75	0.03	0.63	0.43	1.12	0.15	0.02	0.90
Recent exp.(lug = 5)	0.27	0.73	0.01	0.94	0.03	0.97	0.11	0.74
Recent exp. (lug = 10)	0.24	0.75	0.12	0.73	−0.03	0.97	0.41	0.52

^a^ ICD-9 410, ICD-10 I21; ^b^ Units of 100 mg·m^−3^-years for US and UK and 100 unit-years for Germany; ^c^ Age is basic time variable with additional adjustment for year of birth (continuous variable); ^d^ heterogeneity chi^2^ and heterogeneity *p*-value; ^e^ Additional adjustment for plant group (company) in US, plant group in UK, one plant in Germany (no further adjustment); ^f^ weighted by number of expected cases based on national rates (US: 113.2, UK: 97.4, Germany: 40.4) and *p*-value of effect.
